# Enhancing High-Temperature Performance of Flexible Pavement with Plastic-Modified Asphalt

**DOI:** 10.3390/polym16172399

**Published:** 2024-08-24

**Authors:** Salamat Ullah, Ali Qabur, Ansar Ullah, Khaled Aati, Mahmoud Abdelrahim Abdelgiom

**Affiliations:** 1 Key Lab of CAD & CG, Laboratory of Soft Machines and Smart Devices of Zhejiang Province & Department of Engineering Mechanics, Zhejiang University, Hangzhou 310027, China; salamatkhattak@gmail.com; 2 Department of Civil and Architectural Engineering, Jazan University, Jazan 45142, Saudi Arabia; kaati@jazanu.edu.sa (K.A.); mabdelmahmoud@jazanu.edu.sa (M.A.A.); 3 Department of Civil Engineering, Abasyn University, Islamabad 44000, Pakistan; khanansar702@gmail.com

**Keywords:** rutting, modified asphalt, polymer-modified asphalt, rutting resistance, wheel-tracking test

## Abstract

Previous studies indicate that traditional asphalt mixtures lack the ability to withstand the stresses caused by heavy traffic volumes under high temperatures. To enhance the rutting resistance of flexible pavement under high levels of temperature and loading, extensive laboratory experiments were carried out. A 60/70 grade bitumen was used as a neat sample for comparison. The study introduced three distinct polymers, polypropylene (PP), low-density polyethylene (LDPE), and acrylonitrile butadiene styrene (ABS), at varying concentrations by weight into the neat bitumen. Initially, conventional tests were performed to evaluate the conventional properties of both the neat and modified bitumen, while aggregate tests assessed the mechanical properties of the aggregates. Subsequently, a Marshall mix design was performed to determine the optimum bitumen content (OBC) in the asphalt mixture. Finally, wheel-tracking tests were performed under a specific load and temperature to investigate the rutting behavior of the modified asphalt mixtures. The results of this comprehensive study revealed that the modified asphalt mixtures displayed improved resistance to rutting compared to the neat asphalt mixture. Furthermore, it was also observed that the LDPE exhibited a superior performance against rutting, followed by the PP and ABS. At polymer contents of 3%, 5%, and 7%, the LDPE achieved reductions in rut depth of 13%, 24%, and 33%, respectively, outperforming both PP- and ABS-modified asphalt. These findings not only enhance our understanding of asphalt behavior under diverse conditions but also highlight the potential of plastic-modified asphalt as an effective solution for mitigating rutting problems in road pavements. By incorporating plastic modifiers into asphalt mixtures, this approach aligns with the principles of sustainable construction by reducing plastic waste while improving pavement durability and performance.

## 1. Introduction

Flexible pavements commonly encounter issues such as premature road failures, which are evident as permanent deformations, fractures, and surface wear [1]. The primary contributors to these failure issues in asphalt concrete mixtures are escalating traffic volumes, heavy vehicle loads, elevated tire pressures, temperature increases, low-quality materials, and improper asphalt mix designs [2]. Conventional materials prove inadequate for withstanding heavy loads, resulting in the development of rutting on pavements [3].

Researchers are actively engaged in improving the rutting resistance in asphalt. This includes integrating different modifying powders, fibers, and plastic polymers into both the asphalt binder and the asphalt concrete mixture [4]. Previous studies have shown that asphalt mixtures containing additives have a higher strength in withstanding rutting than asphalt mixtures without additives [5]. In recent years, researchers have employed various additives to modify the asphalt binder, aiming to mitigate the issue of early rutting in hot mix asphalt (HMA) [6]. It has also been shown that sample preparation and handling are very important for polymer-modified bitumen, which can affect its rheological properties [7]. Several other recent studies have explored various methods for enhancing the performance of pavement materials. These investigations provide valuable insights into the use of advanced materials and techniques to improve the durability, strength, and overall performance of structures [8,9,10,11,12]. The research in this domain remains vigorous, aiming to enhance the rutting performance of flexible pavement.

This study aims to enhance pavement’s resilience to heavy stresses and high temperatures by incorporating modifiers in both the asphalt binder and asphalt mixture. Within the scope of this research, the asphalt binder undergoes modification with three distinct types of plastic polymers, each applied in varying proportions to enhance its resistance to rutting. The study employs a wheel-tracking performance test to assess the rutting performance of various modified asphalt mixtures. The insights gained from this research are poised to guide pavement engineers in selecting the most appropriate polymer modifier in optimal concentrations, thus contributing to improved pavement performance in the future. The study can be useful for the application of plastic modifiers for different construction purposes.

The following section provides a brief discussion on the utilization of plastic polymer-modified bitumen and polymer-modified asphalt mixtures in asphalt mixtures.

### 1.1. Plastic-Modified Bitumen

A low-density polyethylene (LDPE) is used as a bitumen modifier to improve the behavior of asphalt mixtures. Various tests have been conducted, revealing that LDPE-modified bitumen exhibits reduced levels of penetrability and an elevated softening point compared to pure bitumen [13]. Additionally, incorporating plastic waste into pure bitumen has been observed to decrease the void content of the mix while significantly enhancing its stiffness modulus and water resistance. The use of plastic waste (LDPE) increases its stiffness and elastic behavior and improves its rheological properties, which eventually increase its resistance to aging [14]. Waste packaging polypropylene (WPP)- and WPP/organic rectorite (OREC)-modified asphalt, prepared through melt blending, exhibit improved levels of ductility, plasticity, and high-temperature stability. The optimal OREC content ranges from 1.5% to 2%, with an enhanced performance compared to that of base asphalt [15]. The impact of recycled plastic wastes (RPWs), including polypropylene, high-density polyethylene (HDPE), and LDPE, on the viscoelastic performance of the regional asphalt binder has been explored. The results indicate an improved rutting performance, an increased upper PG limit, and an enhanced resilient modulus. AASHTO design guide modeling predicts significant improvements in pavement distress, highlighting a potential requirement for elastomeric polymer supplementation [16]. Previous investigations revealed the incorporation of polypropylene into bitumen at different proportions (0.5%, 1%, 1.5%, 2%, 2.5%, 3%, and 5% by weight of the binder) through the process of melting bitumen asphalt at temperatures between 153 °C and 159 °C. Experimental findings indicated a marginal reduction in penetration and a slight elevation in the softening point for the modified bitumen when compared to the base bitumen [17]. Their statistical results also demonstrated a slight increase in the softening point and a decrease in penetration grade [18]. It is also noted that adding polypropylene to bitumen decreases its ductility and increases the softening point, which leads to an increase in the stiffness of the binder [19]. The use of waste plastic, such as carrying bags and cups, has been studied as a modifier for asphalt and cement concrete pavement. Various proportions of plastics, including polypropylene, low-density polyethylene, and high-density polyethylene, were mixed with 80/100 paving-grade asphalt. This blending led to an enhanced performance and superior values of the eco-friendly asphalt concrete [20]. Previous studies evaluate the high-temperature performance of tafpack super, tire rubber, and polypropylene-modified bitumen. Using dynamic shear rheometer and wheel-tracking tests, it was concluded that tire rubber exhibits superior high-temperature characteristics and a lower rutting depth compared to those of polypropylene and tafpack super [21]. Numerous researchers have incorporated LDPE in proportions of 2%, 4.5%, and 5% by weight of bitumen at 180 °C. Experimental findings indicate that elevating the LDPE percentage tends to raise the softening point while decreasing levels of ductility and penetration [22]. Other researchers have arrived at a similar conclusion, noting that an increase in LDPE content leads to a reduction in binder penetration. They also observed that enhancing the waste plastic content up to 8% resulted in improved Marshall stability levels for the modified bitumen [23]. In previous research, LDPE was incorporated in various ratios, reaching up to 9% at 170 °C. The statistical outcomes indicated an elevation in the softening point and a reduction in penetration [24]. A similar outcome was affirmed by [25], stating that the addition of LDPE plastic waste increases the softening point while decreasing levels of ductility and penetration. Researchers examined the influence of incorporating locally sourced polyethylene terephthalate (PET) plastic into binder class C320. The binder has a higher viscosity and produces stiffer asphalt, which improves its rutting resistance and mitigates other problems associated with higher temperatures and traffic loads. The assessment of different proportions of PET-modified bitumen was conducted in two stages, considering both unaged and aged conditions. The findings revealed that the optimal content of waste plastic is in the range of 6–8%, effectively enhancing levels of rutting and aging resistance [26].

### 1.2. Plastic-Modified Asphalt Mixture

Significant efforts have been made to improve the mechanical properties of asphalt mixtures using additives and polymers. Researchers have tried to alter the mechanical properties of asphalt mixtures, such as their indirect tensile strength, moisture damage, stiffness, fatigue, cracking, and rutting resistance. Experimental results have shown that adding polymers by a dry process improves their resistance to moisture damage at 15 °C, improves their stiffness at 10, 20, and 40 °C, and lowers their rutting resistance in terms of rut depth at 60 °C [27]. Researchers have investigated the effect of different plastic polymers like PET, PP, polyvinyl chloride (PVC), LDPE, HDPE, and ABS in asphalt mixtures [28]. Some other related studies also discuss the use of modifiers in civil engineering applications [29,30,31,32,33].

Plastic-waste-modified bitumen shows better anti-aging behavior than crumb-rubber-modified bitumen [34]. The use of recycled linear low-density polyethylene (R-LLDPE) in bitumen modified for road applications has been studied. It was found that incorporating R-LLDPE increases its viscosity, softening point, and thermal stability. The study suggests that 3% R-LLDPE is suitable for various environmental conditions, while 6% is recommended for tropical climates, cautioning against higher dosages. Overall, R-LLDPE enhances bitumen performance without significant drawbacks when sourced correctly [35]. Studies indicate that the modification of bitumen with polypropylene enhances its resistance to aging. Additionally, the modified bitumen demonstrated increased levels of elasticity, durability, and adhesion, along with enhanced resistance to rutting and moisture damage [36]. Damping asphalt mixtures have better resistance to rutting than traditional asphalt mixtures due to their lower water sensitivity, necessary mechanical strength, and denser characteristics [37]. Researchers have used low-density polyethylene with 1, 2, and 3% by weight of binder in asphalt to improve the performance of the asphalt. The results suggest that the optimum LDPE concentration is 1% to improve its rutting resistance [38]. Previous studies indicate the use of LDPE in the concentrations of 2.5, 5, 7.5, and 10 by weight to an asphalt binder. An indirect tensile resilient modulus test was carried out on the Humberg wheel tracker. The test result suggests that 5% LDPE is the optimum concentration [39]. Researchers added 2.5, 5, and 15% ABS by weight of the asphalt binder to modify the asphalt’s performance. It was concluded that the 5% ABS concentration gave the optimum result [40]. This study focuses on adding 5% ABS in asphalt by weight of binder. The asphalt binder (bitumen) was heated to 180 °C to 260 °C before mixing it with the ABS polymer. It was concluded that the ABS increased the performance of the asphalt [41]. This research concluded that graphene platelets in asphalt enhance the mechanical properties of asphalt concrete by decreasing the rut depth up to 60% [42]. Mashaan et al. investigated the addition of 8% PET to asphalt, which showed improvement in levels of stability and rut depth by means of an increased Marshall stability and decreased rut depth [43]. When plastic- and rubber-modified asphalt mixtures were investigated under moisture, it was revealed that they could decrease the rutting resistance and elastic modulus by 9% to 17% [44]. Based on the literature, it is concluded that a 5% concentration of LDPE and ABS polymers yielded the most optimal results in terms of overall performance. This concentration was found to strike a balance between enhancing the desired properties of asphalt, such as improved levels of durability and resistance to deformation, without compromising on other critical aspects. Various other studies examine alternative pavement materials and composite materials for improved pavement construction [45,46,47,48,49].

### 1.3. Wheel Tracker

The wheel-tracking test is broadly used to study the rutting performance of asphalt under different temperatures [50]. This study illustrates the use of a wheel tracker and multiple stress creep test apparati to evaluate the rutting performance of different modified asphalts [51]. The Humberg wheel-tracking device test is recommended as the pass/fail criterion during the design phase [52]. An alternate study recommended employing the wheel tracker instead of the asphalt pavement analyzer, as it anticipates resistance to both rutting and moisture damage [53]. Therefore, the Hamburg wheel-tracking test (HWTT) is widely used for assessing asphalt mixtures for rutting and moisture susceptibility [54]. 

### 1.4. Research Gap

The existing research in flexible pavement engineering highlights the prevalence of premature road failures attributed to permanent deformations, fractures, and surface wear, with contributing factors including escalating traffic volumes, heavy vehicle loads, elevated tire pressures, temperature increases, low-quality materials, and improper asphalt mix designs. Despite ongoing efforts to enhance rutting resistance in asphalt, gaps persist in our understanding of the optimal integration of modifying agents such as powders, fibers, and plastic polymers into asphalt binders and mixtures. While previous studies demonstrate the efficacy of additives in improving asphalt mixture strength and rutting resistance, there is limited exploration into the simultaneous modification of both asphalt binders and mixtures to bolster pavements’ resilience to heavy stresses and rising temperatures. Moreover, research addressing the influence of sample preparation and handling on the rheological properties of polymer-modified bitumen remains sparse. Consequently, there is a pressing need for comprehensive investigations to systematically evaluate the performance of different modifier types (which have not been explored yet) and proportions in enhancing rutting resistance and overall pavement durability. This study seeks to address these gaps by examining the impact of three distinct plastic polymer modifiers on asphalt binders and mixtures, utilizing wheel-tracking performance tests to assess rutting resistance. The insights gathered are expected to provide valuable guidance to pavement engineers in selecting appropriate polymer modifiers and concentrations, thereby contributing to the advancement of pavement performance and durability against rutting.

Research in this domain remains vigorous, aiming to enhance the rutting performance of flexible pavement. Within the scope of this research, the asphalt binder undergoes modifications with three distinct types of plastic polymers, each applied in varying proportions to enhance its resistance to rutting. The study employs a wheel-tracking performance test to assess the rutting performance of various modified asphalt mixtures. The insights gained from this research are poised to guide pavement engineers in selecting the most appropriate polymer modifier in optimal concentrations, thus contributing to improved pavement performance in the future.

## 2. Materials and Methods

**Figure 1:**
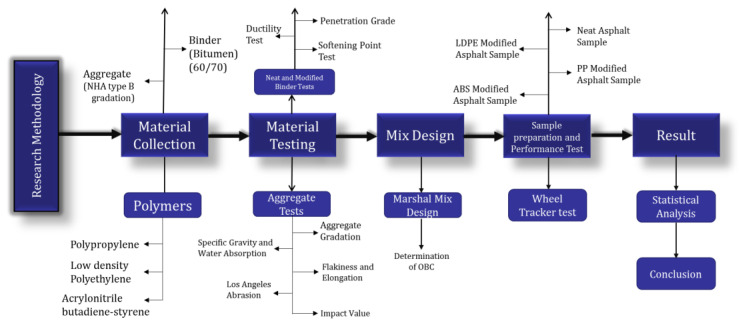
Flow chart: research methodology.

An outline of the research methods adopted for this study is shown in Figure 1.

### 2.1. Materials

The method involves utilizing three polymers, namely PP (molecular weight: 315,000 g/mol, melting point: 184 °C, density: 0.934 ± 0.005 g/cm^3^), LDPE (molecular weight: 111,500 g/mol, melting point: 120 °C, density: 0.92 g/cm^3^ ± 0.01), and ABS (molecular weight: 95,000 g/mol, melting point = 105 °C ± 5 °C, density: 1.05 g/cm^3^, styrene: 50%, acrylonitrile: 25%, butadiene: 25%) as modifiers for asphalt. These polymers are abundantly available in Pakistan and easy to handle. PP and LDPE are categorized as thermoplastic polyolefin polymers. A total of ten samples of modified asphalt binders are utilized in the making of asphalt mixtures. In this study, 3%, 5%, and 7% plastic contents are selected based on the existing literature, which demonstrates the effect of these doses on the properties of bitumen. These studies concluded that mixing these plastic polymers in bitumen will improve the rheological properties of the binder, which is essential for the rutting resistance of asphalt mixtures [55]. Also, some studies indicate the negative impact of high plastic content, which also contributes to the selection of plastic dosages [55,56]. These samples include one unmodified (neat) bitumen sample and nine modified bitumen samples derived from PP, LDPE, and ABS.

Hot mix asphalt is typically prepared with different sizes of aggregates mixed with mineral fillers and is uniformly mixed and coated with asphalt cement. Each has unique characteristics that make it more suitable for design and construction purposes. Aggregates for the current study were obtained from Margalla quarry, near Islamabad, which is widely used in Pakistan. Plastic polymer is obtained from a local plastic recycling plant near Islamabad.

### 2.2. Aggregate Properties

Size and aggregate grading is directly regulated by NHA (aggregate grade “class-B” for wearing coarse; NHA General Specification, 1998). NHA, in its general specifications, has specified two aggregate gradations, namely class ‘A’ and ‘B’, which are the coarser one and finer one, respectively. Gradation of NHA class-B has arbitrarily been selected for this study for all asphalt mixture samples as it is commonly used in the field, as reported in Table 1.

Aggregate conventional tests were conducted on aggregate to verify that the properties of aggregate used in the study fall under the prescribed allowable limit. The physical properties of aggregate are shown in Table 2 below.

### 2.3. Experimental Program

This study is divided into three stages. The first stage is based on the modification of bitumen and the change that occurs in its conventional properties by adding modifiers is studied. In the second stage, a Marshall mix design test is conducted to investigate the effect of polymer type and optimum bitumen content (OBC). Finally, a wheel-tracking test is carried out on asphalt mixtures to study the rutting performance of plastic-modified asphalt (PMA).

### 2.4. Sample Preparation

**Figure 2:**
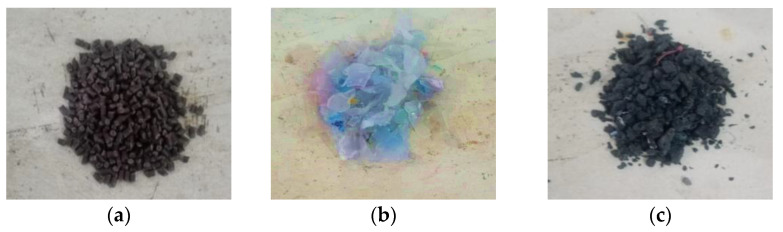
(**a**) PP, (**b**) LDPE, (**c**) ABS.

As mentioned, one neat bitumen sample (0% polymers) and nine modified bitumen samples as binders are used in this study. In this study, the bitumen of pen grade 60/70 was modified with three types of polymers: PP, LDPE, and ABS, as shown in Figure 2. These polymers are mixed with neat bitumen with contents of 3%, 5%, and 7% by weight. Firstly, bitumen was heated to about 150 °C until it melted completely. The subsequent mixing of bitumen with polymers occurs at a temperature of around 180 °C, with a tolerance of ±5 °C. This process ensures that the bitumen–polymer blend reaches the desired consistency and performance characteristics.

Lastly, the prepared modified bitumen blend was stored in a container for further testing. After preparing modified bitumen blends, the bitumen is subjected to conventional testing, including tests of its levels of penetration, softening point, and ductility, to investigate the changes that occur due to the addition of polymers.

### 2.5. Marshall Mix Design

**Figure 3:**
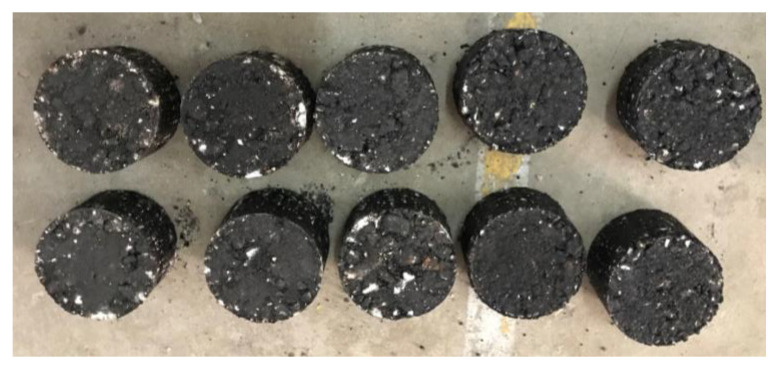
Compacted samples of Marshall stability.

Optimum bitumen content in asphalt mixture is considered a primary factor. Therefore, it is very important to determine the accuracy of the OBC for asphalt mixture samples. A Marshall mix design method following ASTM D1559 [63] was used to determine the OBC for modified and unmodified samples. In this method, 10 compacted samples of 3.5%, 4%, 4.5%, 5%, and 5.5% bitumen were prepared by using a standard Marshall hammer for modified and unmodified bitumen. According to Asphalt Institute requirements, a normal hammer weighing 2.04 kg was dropped from a height of 0.457 m for 75 blows on each side of the sample in Marshall mold. Five loose samples were also prepared for each type of asphalt mixture to determine the maximum specific gravity by following ASTM D2041 [64]. Then, these samples were subjected to Marshall stability and flow tests. Figure 3 shows the samples.

### 2.6. Wheel Tracking

**Figure 4:**
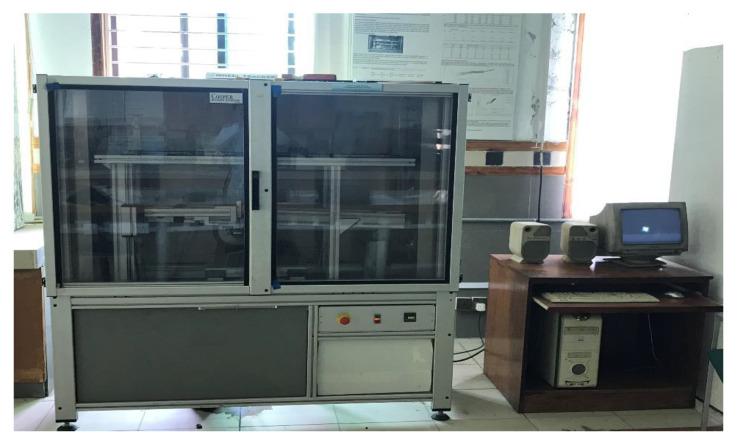
Cooper wheel tracker.

In this study, the Cooper wheel tracker manufactured by cooper technology is used to investigate the rutting performance of various modified PMA and unmodified asphalt mixtures. The sample used was a slab specimen of 150 mm × 150 mm × 50 mm. The weight of the materials needed was estimated based on the volume of the mold and the specific gravity of asphalt ($G_{mb}$). The required weight of aggregate with the OBC of bitumen was mixed under 150 °C. Cooper roller compactor was used to compact the sample under standard pressure and passes following EN 12697-33 [65] standard. Then, after cooling the sample down to room temperature for 24 h, samples were subjected to a Cooper wheel-tracking test under 700 N loads, 55 °C temperature, and 10,000-wheel passes. Results were directly observed and recorded in the attached computer with the machine and were collected in a text file for further analysis. Figure 4 shows the Cooper wheel-tracking machine. 

## 3. Results and Discussion

### 3.1. Penetration Test

The penetration test is one of the major tests for bitumen. The test was conducted by following the ASTM D5/AASHTO T49 [66,67] procedure. We use 60/70 pen-grade bitumen, which was also confirmed by a penetration test conducted on the neat bitumen. The results of the penetration tests are shown in Table 3.

As shown in Table 3, the penetration of the modified bitumen decreases with increasing percentages of the polymer content. The result shows an improvement in the toughness of bitumen with the addition of plastic polymers. Figure 4 shows the trends of the penetration of the modified bitumen.

**Figure 5:**
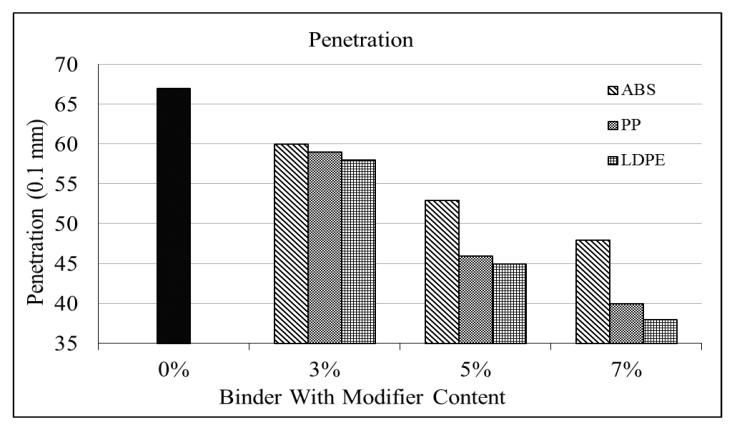
Penetration vs. % binder.

As seen from Figure 5, the penetration of PP-modified bitumen decreases with increments in the percentage of the PP dosage. It is observed that with every 3% increment in PP content, the level of penetration decreases by an average of 16%. Moreover, the minimum penetration level of 4.0 mm (a 40% decrease w.r.t to base bitumen) occurs at the 7% level of PP content.

The performance of the LDPE in terms of penetration was better than the performance of the other two polymers. The LDPE shows the minimum level of penetration, which is 3.8 mm at the 7% level of LDPE content. There is an average decrease of 17% for every 3% increment in LDPE content. 

The ABS shows the same trend as the PP and LDPE, but its decremental average is less than that of the other two polymers. The ABS shows an 11% decrease in penetration as compared to decreases of 16% and 17% for the PP and LDPE, respectively.

All three polymers have the same penetration trend of a gradual decrease in pen grade value. Figure 5 shows, however, that the LDPE has the lowest penetration level for each of the three percentages. The LDPE-modified bitumen can be used for heavy-loaded pavements because of its toughness.

### 3.2. Softening Point

The softening point is another important characteristic of bitumen used to predict its performance. The test was carried out using the standard procedure of ASTM D36/AASHTO T53 [52,53] and the ring and ball apparatus. The results of the softening point tests are shown below in Table 4.

**Figure 6:**
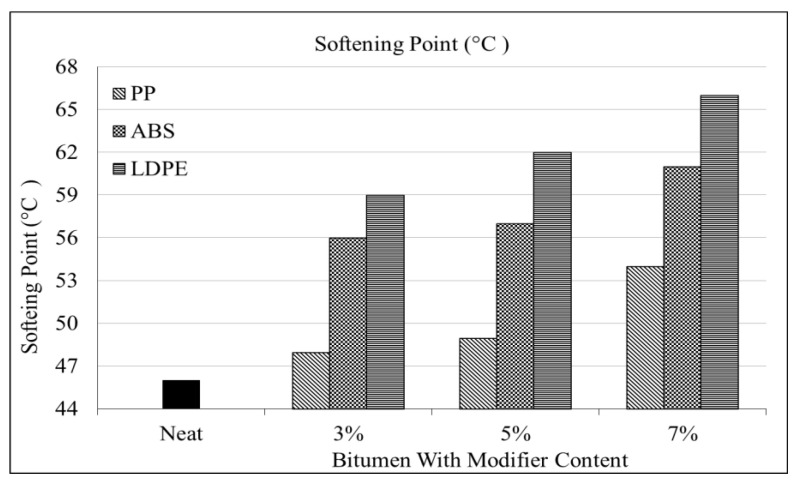
Softening point vs. % polymer.

The results show an increase in the softening point of bitumen as the polymer content increases. Figure 6 below represents the softening trends. 

Figure 6 shows that the softening point increases with increasing PP percentages. The PP shows 4%, 7%, and 17% increases at the 3%, 5%, and 7% PP polymer contents. The maximum value was at the 7% level of PP content, which is (54 °C) lower than the other two polymers.

After the penetration, the LDPE also showed the best results for the softening point, as shown in Figure 6. The LDPE shows a 43% increase in the softening point of bitumen at the 7% dosage level as compared to 17% and 33% increases for PP and ABS. With a 7% rise in ABS content, the softening point experiences a 33% increase. The result obtained from the ring and ball apparatus shown in Table 5 and Figure 6 shows an increase in the softening point of the polymer-modified binder with respect to the base bitumen. 

### 3.3. Ductility

The ductility test is used to evaluate the resistance of bitumen against cracking and rupture. This test was carried out following the ASTM D113 [68] standard procedure. Table 5 contains the results of the ductility testing machine.

**Figure 7:**
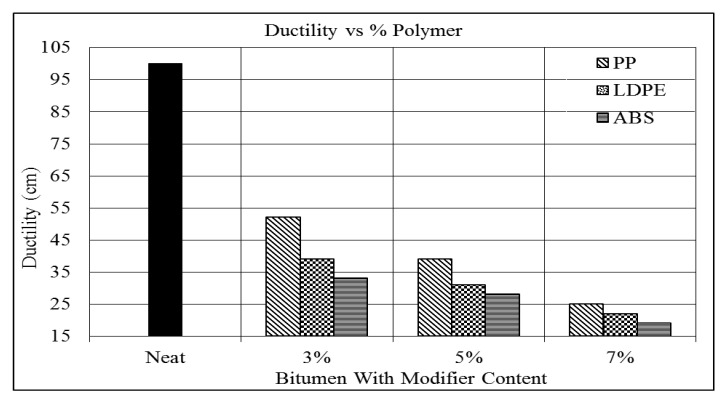
Ductility vs. % polymer.

As seen from Table 5 above, the ductility of bitumen decreases with the increasing polymer content. The trends of the results are shown graphically in Figure 7.

Figure 7 shows that the LDPE ductility of the modified bitumen decreases with an average of 11% for each percentage of LDPE added to the bitumen binder. Also, it can be seen that at the 7% level of LDPE content, the ductility is 22 cm compared to the base bitumen’s ductility of 100 cm.

The ductility of ABS-modified bitumen is lower than that of the other modifiers. The ABS shows 6% less ductility than the PP and 3% less than the LDPE. The minimum ductility is 19 cm at the 7% level of ABS, as shown in Figure 7, as compared to 25 cm and 22 cm for the PP and LDPE, respectively.

The result obtained from the ductility machine shown in Table 5 and Figure 7 shows the rapid decrease in ductility of the polymer-modified binder with respect to that of the base bitumen. Ductility tests provide an easy way to determine the quality of bitumen by measuring its resistance to plastic deformation and cracking. With the addition of polymers, bitumen gets harder and stiffer, and a reduction in ductility is predicted.

### 3.4. Viscosity Test

The viscosity test is conducted to determine the mixture viscosity by incorporating different percentages of polymers. The viscosity of the bitumen is measured using a rotational viscometer at a test temperature of 135 °C, with PP, LDPE, and ABS added at different concentrations. The purpose of the viscosity test is to evaluate the workability of the mixture. A high viscosity can significantly hinder the ability of the mixture to thoroughly blend with the aggregate [69]. This is crucial in ensuring the proper coating and bonding of the bitumen with the aggregate, which are essential for the durability and performance of the asphalt pavement. The Strategic Highway Research Program (SHRP) stated a maximum viscosity of 3 Pa·s at 135 °C for workability purposes [70]. The results, as shown in Table 6, indicate a clear trend: the viscosity of the bitumen increases with the increase in polymer content. This finding highlights the significant influence of the addition of polymers on the rheological properties of bitumen, thereby providing valuable insights into optimizing its formulation for enhanced performance in various applications. 

### 3.5. Types of Modifiers

The second stage of this study investigated the effect of the polymers on the PMA mixtures’ OBC. The results of the Marshall mix design and a discussion of the PMA mixture are given separately in this section. The OBC was determined by following ASTM standard D1559 [63].

#### 3.5.1. Polypropylene (PP)

The OBC was determined after determining the Marshall properties of the PP-modified asphalt. Table 7 contains the Marshall properties, including the OBC, of the PP-modified asphalt along with the neat asphalt mixture.

From Table 7 above, it is evident that the increase in PP content affects the stability and OBC of the asphalt mixture. However, after 3%, the stability of the PP-modified asphalt mixture did not increase significantly. 

**Figure 8:**
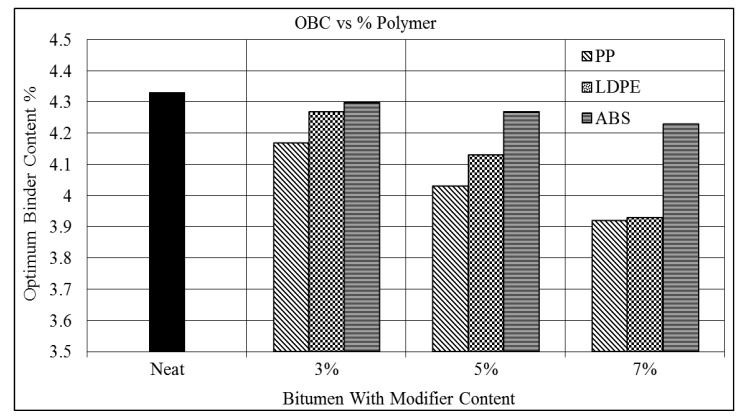
OBC vs. % modifier.

Figure 8 shows the relation between the OBC and modifier content. It shows that the OBC decreases with increasing percentages of the PP polymer. It is observed that by modifying the asphalt with the PP, the OBC decreased by 4%, 7%, and 9% when 3%, 5%, and 7% levels of PP were added. The minimum OBC of 3.92 occurs at the 7% level of PP as compared to 4.33 of the neat asphalt.

Table 8 shows that a decreasing trend in the OBC was observed when the percentage of LDPE increased. As compared to the PP-modified asphalt, the LDPE shows a lower improvement in OBC. At the 3% modification level of the PP and LDPE, the OBC decreased by 4% and 1%. However, at the 7% modification level, both the PP and LDPE show the same 9% decrement in OBC. Table 9 shows that the ABS-modified asphalt did not demonstrate a significant improvement in the optimum binder content. Only a 1% reduction is seen in the OBC for the 3% ABS-modified asphalt mixture. The OBCs of the 3%, 5%, and 7% modified asphalt specimens were 4.3, 4.27, and 4.23 as compared to the OBC of 4.33 for the neat asphalt mixture.

HMA pavement must be designed with an optimal binder content to achieve required levels of durability, strength, and performance. OBC also affects the overall cost of constructed pavements. As observed above, the polymers positively affect the OBC of the asphalt mixture. It can be observed from the figure that the 3% modification level of the asphalt mixture with PP, LDPE, and ABS results in optimum binder contents of 4.17, 4.27, and 4.3. These indicate that at 3%, the PP performs better than the LDPE and ABS, as the PP results in a 4% lower OBC than the neat asphalt mixtures, compared to 1% of the LDPE and ABS.

Figure 8 shows that the OBC at the 5% addition level of polymers also has the same trend as the 3% modified asphalt mixture. If the asphalt mixture were modified with 5% plastic polymer, then the PP-modified asphalt would still perform better than the LDPE and ABS. The PP results in 3% and 7% lower OBCs compared to those of the LDPE and ABS. It is concluded that the PP and LDPE result in almost the same OBC, which means they both perform better than the ABS. The PP and LDPE result in the same OBC, which is 9%, as compared to ABS, which has an OBC of 2%.p

#### 3.5.2. Low-Density Polyethylene (LDPE)

The Marshall properties of the LDPE-modified asphalt mixture at the OBC are shown in Table 8 below.

**Table 8:** Marshall mix properties of neat and LDPE-modified asphalt mixture.

Sr. No	% Polymer	OBC (%)	Stability (kg)	Unit Weight (kg/m^3^)	Flow (0.25 mm)
0	Neat	4.33 ± 0.067	1030	2342	8
1	3% LDPE	4.27 ± 0.13	2125	2200	7
2	5% LDPE	4.13 ± 0.06	2126	2126	6.9
3	7% LDPE	3.93 ± 0.087	2130	2130	6.5

#### 3.5.3. Acrylonitrile Butadiene Styrene (ABS)

The Marshall properties of the ABS-modified asphalt mixture at the OBC are shown in Table 9 below.

**Table 9:** Marshall mix properties of neat and ABS-modified asphalt mixture.

Sr. No	Description	OBC (%)	Stability (kg)	Unit Weight (kg)	Flow
0	Neat	4.33 ± 0.067	1030	2342	8
1	3% ABS	4.3 ± 0.03	2100	2102	7.2
2	5% ABS	4.27 ± 0.033	2113	2113	7.4
3	7% ABS	4.23 ± 0.088	2120	2219	7.6

### 3.6. Wheel Tracker

**Figure 9:**
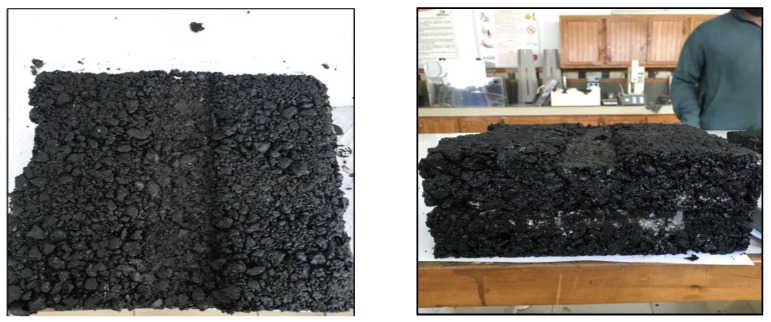
Slab specimens in wheel-tracking tests.

**Figure 10:**
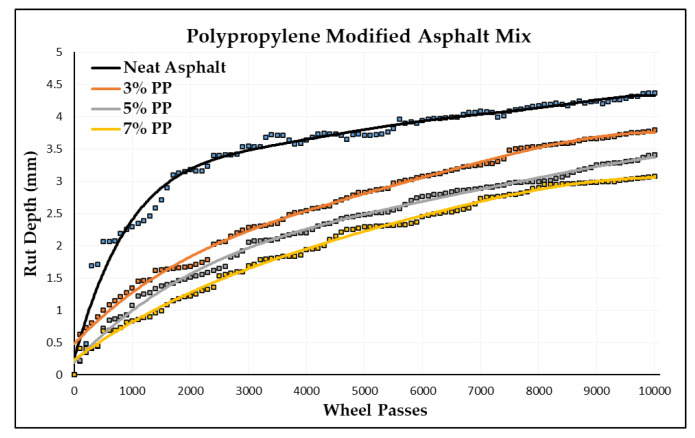
Rut depth vs. wheel passes (PP-modified).

In this section, permanent deformations in terms of rut depth are measured by a wheel-tracking device. The Cooper wheel-tracking machine test was used to determine the rut depth of one neat asphalt and nine PMA specimens by following the EN 12697-22 [71] standard. Figure 9 shows some specimens from the wheel-tracking tests. The rut depth for each number of passes is recorded automatically in the software by a computer attached to the device. These data were acquired from the software, and the graph between the rut depth and number of passes was plotted for each type of asphalt specimen. Table 10 shows the rut depth at 10,000 passes of different polymer-modified asphalt specimens, and Figure 10 shows the slope between the rut depth and number of passes.

## 4. Discussion

Table 10 provides a comprehensive overview of the results obtained from the wheel-tracking tests conducted on the various asphalt mixtures, including both the neat and modified formulations. The data reveal that the neat asphalt mixture exhibited a rut depth of 4.37 mm after 10,000 passes. In comparison, the three modified asphalt mixtures exhibited varying levels of rut depth, indicating their respective performances in resisting rut formation. Specifically, at a 3% incorporation rate, a low-density polyethylene (LDPE) asphalt mixture demonstrated an improved performance with a rut depth of 3.79 mm, representing a notable 13% enhancement in rut resistance compared to that of the neat asphalt mixture. Similarly, at a 5% incorporation rate, a modified LDPE asphalt mixture exhibited an enhanced performance with a rut depth of 3.30 mm, showcasing a significant 24% improvement in rut resistance compared to that of the neat asphalt mixture. Furthermore, at a 7% incorporation rate, an LDPE asphalt mixture displayed a superior performance with a rut depth of 2.91 mm, indicating a remarkable 33% improvement in rut resistance relative to that of the neat asphalt mixture.

These findings highlight the effectiveness of incorporating polymer modifiers, such as PP, LDPE, and ABS, into asphalt mixtures to enhance their rut resistance properties. The observed reductions in rut depth underscore the potential of these modifiers to mitigate the damaging effects of repeated loading and traffic-induced stresses on asphalt pavements. Notably, the highest improvement in rut resistance was achieved with a 7% incorporation rate of LDPE, suggesting the potential for the further optimization of modifier concentrations to achieve even greater enhancements in pavement performance. Overall, these results contribute valuable insights into the optimization of asphalt mixture formulations for the enhanced durability and longevity of flexible pavements. 

Figure 10 shows the relation between the wheel passes and rut depth of an unmodified or neat asphalt mixture. It can be observed that by increasing the number of wheel passes, the rut depth increases and vice versa. The maximum rut depth of 4.37 mm can be seen at 10,000 passes.

An analysis of the results obtained from the cooper wheel tracker tests revealed a notable trend: as the polymer content in the asphalt mix increases, there is a corresponding enhancement in its resistance to rutting. This observation is depicted in Figure 10, in which it can be observed that the rut depth of the asphalt mixture modified with PP decreases as the polymer content increases. Specifically, the addition of PP results in an improvement in its rut resistance by 13%, 20%, and 30% at polymer content levels of 3%, 5%, and 7%, respectively, when compared to the neat asphalt mixture. Notably, the lowest rut depth recorded was 3.07 mm, which was achieved by a 7% PP-modified asphalt mixture. These findings underscore the efficacy of PP in mitigating rutting and highlight its potential to enhance the performance of asphalt mixtures under various loading conditions.

**Figure 11:**
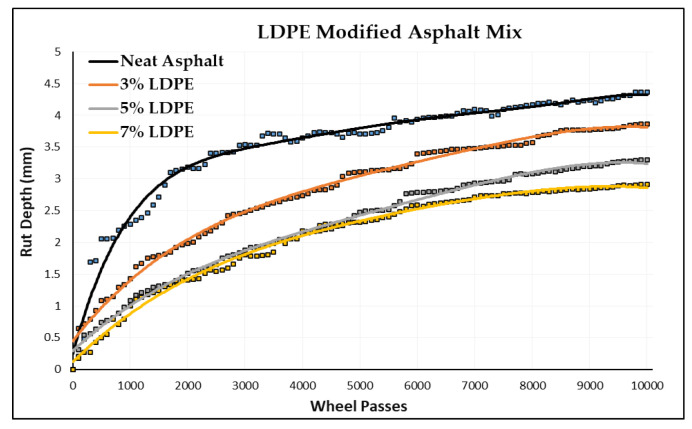
Rut depth vs. wheel passes (LDPE-modified).

LDPE-modified asphalt likewise exhibits a robust level of resistance to rutting, showcasing notable improvements in rut resistance, particularly at a 7% LDPE concentration, as shown in Figure 11. This enhancement is evidenced by a substantial 33% reduction in rutting compared to that of unmodified asphalt specimens. Specifically, the incorporation of LDPE results in rut depths of 3.86 mm, 3.3 mm, and 2.91 mm at LDPE addition rates of 3%, 5%, and 7%, respectively, within the asphalt mixture. These findings underscore the effectiveness of LDPE in strengthening the durability and performance of asphalt mixtures, with implications for mitigating rutting-related deterioration and ensuring long-term pavement integrity.

**Figure 12:**
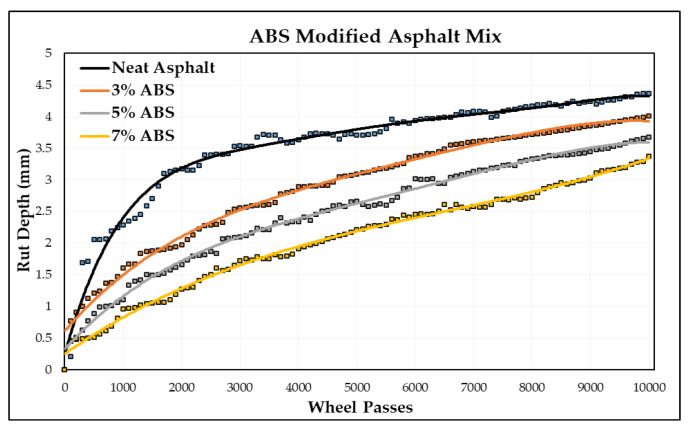
Rut depth vs. wheel passes (ABS-modified).

According to the data presented in Figure 12, there is a noticeable trend of decreasing rut depths with an increase in ABS content within the asphalt mixture. However, it is important to note that ABS exhibits a comparatively lower level of resistance to rutting when compared to PP and LDPE modifiers. Despite this, the ABS still contributes to an improvement in rutting resistance, although to a lesser extent. Specifically, at ABS content levels of 3%, 5%, and 7%, there are observed increases in rutting resistance levels of 8%, 16%, and 23%, respectively. These findings highlight the varying effects of different modifiers on asphalt performance and underscore the importance of selecting the most appropriate modifier based on specific project requirements and environmental conditions.

There is evidence indicating that at a polymer content of 3%, the LDPE-modified asphalt achieves the lowest rut depth recorded, measuring at 3.79 mm, when compared to that of the asphalt modified with ABS and LDPE. The findings underscore the efficiency of LDPE as a modifier in enhancing the durability and performance of asphalt pavements, offering valuable insights for the selection of appropriate modifiers to optimize pavement performance under various loading and environmental conditions.

At a 5% modification level within the asphalt mixture, LDPE emerges as a high performer, yielding the lowest rut depth recorded at 3.3 mm. LDPE represents a substantial 24% enhancement in the rut resistance level compared to that of the unmodified base asphalt mixture. Remarkably, the LDPE outperforms both the PP and ABS in terms of its rut resistance at the 5% content level. These findings underscore LDPE’s efficacy as a modifier in significantly improving the resilience and durability of asphalt pavements.

The findings emphasize the significant improvements in rut resistance that can be attributed to LDPE, especially when utilized at the 7% polymer content level. Specifically, at this concentration, LDPE demonstrates clear advantages over alternative polymer modifiers. A direct comparison between LDPE-modified asphalt and PP-modified asphalt reveals a notable 5% reduction in rut depth, illustrating LDPE’s superior ability to minimize surface deformations resulting from repeated traffic loads. Moreover, in contrast to ABS-modified asphalt, LDPE displays a considerable 15% increase in rutting resistance levels. These results underscore LDPE’s effectiveness in enhancing the durability and longevity of asphalt pavement, thus playing a crucial role in advancing the sustainability and efficiency of road infrastructure.

## 5. Conclusions

The main focus of this research is to better understand the properties and performance of different modified bitumen types in order to enhance the rutting resistance of flexible pavement under high temperatures and heavy loading conditions. To achieve this objective, extensive laboratory experiments were carried out. These experiments aimed to simulate real-world conditions and assess how different modifications of bitumen could contribute to an improved resistance to rutting. The following conclusions are derived from the outcomes of the conventional tests we conducted on modified bitumen:

1. Utilizing ABS, PP, and LDPE as bitumen modifiers enhances its rheological properties, as shown by reduced ductility and penetration levels, and a higher softening point. The results suggest that higher polymer concentrations make bitumen harder and stiffer;
2. All three polymers show a consistent trend of decreasing penetration values, with LDPE consistently having the lowest penetration value among the three modifiers;
3. The ductility test shows a significant drop in ductility for the polymer-modified binders compared to neat bitumen. Adding polymers makes the bitumen harder and stiffer, reducing its ductility. PP had the highest level of ductility among the polymers tested.

The following conclusions are drawn from the Marshall mix design tests:

1. As the percentage of plastic polymer increases, the optimal binder content for the asphalt mixtures decreases, while the level of stability almost doubles for all types of the plastic-modified asphalt samples;
2. In comparison to PP-modified asphalt, LDPE demonstrates a lower optimum binder content (OBC). ABS-modified asphalt exhibits an even lower OBC;
3. Overall, PP and LDPE perform better than ABS-modified asphalt in terms of OBC. A decrease in OBC is a positive outcome, as it reduces the overall cost of pavement construction and leads to more sustainable pavement solutions.

The following conclusions are drawn from the wheel-tracking test:

1. Adding polymers to the asphalt mixture improves its rutting resistance, with higher percentages of plastic polymer providing better results compared to neat asphalt after 10,000 wheel passes;
2. Modifying asphalt with a 3% polymer content shows that LDPE-modified asphalt has the highest level of rutting resistance, outperforming that of unmodified asphalt, as well as PP and ABS-modified asphalt;
3. At a 7% polymer content level, the findings highlight significant improvements in rut resistance: ABS reduces rutting by 23%, PP by 30%, and LDPE by 33% compared to unmodified asphalt mixtures.

## 6. Recommendations

- Perform wheel-tracking tests, increasing the number of wheel passes to assess durability;
- Enhance bitumen by raising the polymer content, incorporating a combination of all three types of plastic to optimize the overall blend;
- Examine the behavior of these modified asphalt mixtures under various stress conditions to evaluate their performance improvements.

## Figures and Tables

**Table 1:** Selected aggregate gradations.

Sieve Size	Specification	Selected Gradation	
(mm)	NHA Class-B (%)	Percentage Passing (%)	Percentage Retained (%)
19	100	100	0.00
12.5	75–90	82.5	17.5
9.5	60–80	70	12.5
4.75	40–60	50	20
2.38	20–40	30	20
1.18	5–15	10	20
0.075	3–8	5	5
Pan	----	----	5

**Table 2:** Aggregate conventional test result.

Sr. No.	Test Description	Test Specification	Result	Standard Limits
1	Los Angeles Abrasion Value	AASHTO T96 and ASTM C131 [57,58]	21.46%	<30%
2	Water absorption	ASTM C128 [59]	0.367%	<0.6%
3	Specific gravity	ASTM C127 [60]	2.44	2.4–3.0
4	Impact value test	ASTM D5874 [61]	13.80%	<15%
5	Elongation index	ASTM D4791 [62]	7.20%	Max 20%
6	Flakiness index	ASTM D479 [62]	5.82%	Max 20%

**Table 3:** Penetration test results.

Polymer Added in Bitumen by Weight (%)	Penetration (0.1 mm) at 25 °C					
	PP		ABS		LDPE	
	Mean	SE	Mean	SE	Mean	SE
Neat	67					
3	59	±1.851	60	±0.946	58	±1.45
5	46	±1.827	53	±1.154	45	±0.882
7	40	±1.816	48	±1.816	38	±0.577

**Table 4:** Softening point test result.

Polymer Added in Bitumen (%)	Softening Point (°C)					
	PP		ABS		LDPE	
	Mean	SE	Mean	SE	Mean	SE
Neat	46					
3	48	±1.00	56	±0.55	59	±0.42
5	49	±1.18	57	±0.34	62	±1.7
7	54	±0.57	61	±0.75	66	±0.39

**Table 5:** Ductility test result.

Polymer Added in Bitumen (%)	Ductility (cm) at 25 °C					
	PP		ABS		LDPE	
	Mean	SE	Mean	SE	Mean	SE
Neat	100					
3	52	±2.39	33	±0.88	39	±0.33
5	39	±2.33	28	±0.65	31	±0.63
7	25	±0.72	19	±0.55	22	±0.89

**Table 6:** Viscosity test results.

Polymer Added in Bitumen (%)	Viscosity at 135 °C (Pa·s)					
	PP		ABS		LDPE	
	Mean	SE	Mean	SE	Mean	SE
Neat	0.62 ± 0.11					
3	1.17	±0.13	1.14	±0.08	1.34	±0.1
5	1.70	±0.09	1.34	±0.06	1.99	±0.1
7	2.51	±0.07	1.65	±0.10	2.26	±0.12

**Table 7:** Marshall mix properties of neat and pp-modified asphalt mixture.

Sr. No	Description	OBC (%)	Stability (kg)	Unit weight (kg/m^3^)	Flow (0.25 mm)
0	Neat	4.33 ± 0.067	1030	2342	8
1	3% PP	4.17 ± 0.1	2110	2290	7.1
2	5% PP	4.03 ± 0.09	2125	2195	7.08
3	7% PP	3.92 ± 0.06	2158	2170	7.1

**Table 10:** Wheel-tracking test results.

Description		Rut Depth at 10,000 Passes	Percentage Improved (w.r.t. Neat Asphalt)
Polymer Used	Percentage Added		
Neat	0	4.37 mm	
PP	3	3.86 mm	12
PP	5	3.41 mm	22
PP	7	3.07 mm	30
ABS	3	4.01 mm	8
ABS	5	3.68 mm	16
ABS	7	3.37 mm	23
LDPE	3	3.79 mm	13
LDPE	5	3.30 mm	24
LDPE	7	2.91 mm	33

## Data Availability

The original contributions presented in the study are included in the article; further inquiries can be directed to the corresponding author.
